# Structurally Similar Allosteric Modulators of α7 Nicotinic Acetylcholine Receptors Exhibit Five Distinct Pharmacological Effects*[Fn FN2]

**DOI:** 10.1074/jbc.M114.619221

**Published:** 2014-12-16

**Authors:** JasKiran K. Gill-Thind, Persis Dhankher, Jarryl M. D'Oyley, Tom D. Sheppard, Neil S. Millar

**Affiliations:** From the ‡ Department of Neuroscience, Physiology & Pharmacology, University College London, London, WC1E 6BT, United Kingdom and; the § Department of Chemistry, University College London, London, WC1H 0AJ, United Kingdom

**Keywords:** Cys Loop Receptor, Membrane Protein, Molecular Pharmacology, Neurobiology, Nicotinic Acetylcholine Receptors (nAChR), Receptor Structure-Function

## Abstract

Activation of nicotinic acetylcholine receptors (nAChRs) is associated with the binding of agonists such as acetylcholine to an extracellular site that is located at the interface between two adjacent receptor subunits. More recently, there has been considerable interest in compounds, such as positive and negative allosteric modulators (PAMs and NAMs), that are able to modulate nAChR function by binding to distinct allosteric sites. Here we examined a series of compounds differing only in methyl substitution of a single aromatic ring. This series of compounds includes a previously described α7-selective allosteric agonist, *cis-cis*-4-*p*-tolyl-3a,4,5,9b-tetrahydro-3*H*-cyclopenta[c]quinoline-8-sulfonamide (4MP-TQS), together with all other possible combinations of methyl substitution at a phenyl ring (18 additional compounds). Studies conducted with this series of compounds have revealed five distinct pharmacological effects on α7 nAChRs. These five effects can be summarized as: 1) nondesensitizing activation (allosteric agonists), 2) potentiation associated with minimal effects on receptor desensitization (type I PAMs), 3) potentiation associated with reduced desensitization (type II PAMs), 4) noncompetitive antagonism (NAMs), and 5) compounds that have no effect on orthosteric agonist responses but block allosteric modulation (silent allosteric modulators (SAMs)). Several lines of experimental evidence are consistent with all of these compounds acting at a common, transmembrane allosteric site. Notably, all of these chemically similar compounds that have been classified as nondesensitizing allosteric agonists or as nondesensitizing (type II) PAMs are *cis-cis*-diastereoisomers, whereas all of the NAMs, SAMs, and type I PAMs are *cis-trans*-diastereoisomers. Our data illustrate the remarkable pharmacological diversity of allosteric modulators acting on nAChRs.

## Teaser

**Background:** Nicotinic receptors are activated by acetylcholine and have been implicated in several neurological disorders.

**Results:** Allosteric modulators, sharing close chemical similarity, exhibit five distinct pharmacological effects on α7 nicotinic receptors.

**Conclusion:** Small changes in chemical structure have profound effects on the pharmacological properties of allosteric modulators.

**Significance:** These findings may provide opportunities for novel approaches to therapeutic drug discovery.

## Introduction

Nicotinic acetylcholine receptors (nAChRs)^5^ are cation-selective ion channels that belong to the Cys loop superfamily of neurotransmitter-gated ion channels (1, 2). In common with other Cys loop receptors, nAChRs are pentameric complexes in which five transmembrane subunits co-assemble to form a central ion channel (2, 3). Although all nAChRs share a similar three-dimensional structure (4, 5), there is considerable diversity in their subunit composition. For example, in mammalian species, there are 16 nAChR subunits (α1–α7, α9, α10, β1–β4, γ, δ, and ϵ) that can co-assemble to generate a diverse family of nAChR subtypes (6, 7). In addition, some nAChR subunits (such as α7) form functional homomeric nAChRs, containing five copies of the same subunit (8). The human α7 nAChR has been identified as a potential target for therapeutic drug discovery and has been implicated in a number of neurological and psychiatric disorders (9–13). The α7 nAChR is somewhat atypical of this receptor family, in that it undergoes very rapid desensitization in response to activation by its endogenous neurotransmitter acetylcholine (8). However, allosteric modulation can facilitate activation of α7 nAChRs with only low levels of desensitization (14–17).

Activation of nAChRs and the opening of the cation-selective pore is associated with the binding of agonists such as acetylcholine to an orthosteric binding site located in the extracellular domain of the receptor at the interface between two adjacent subunits (18, 19). In addition, a range of compounds have been identified that can modulate nAChR activation by binding to distinct allosteric sites (20) and may have potential as novel approaches to therapeutic drug discovery (21). An area that has attracted considerable interest concerns compounds that lack intrinsic agonist activity on nAChRs but are able to potentiate agonist-evoked responses by binding to a distinct allosteric site. Such compounds have been described as positive allosteric modulators (PAMs) and include compounds such as TQS, a PAM that displays selectivity for α7 nAChRs (15). In the case of rapidly desensitizing nAChRs such as α7, PAMs have been classified as being either type I or type II, depending on whether they cause little or no effect on the rate of agonist-induced desensitization (type I PAMs) or cause a reduction in desensitization (type II PAMs) (20, 22, 23). In addition, there is evidence that the binding of ligands to allosteric sites on nAChRs can result in efficient activation of nAChRs in the absence of orthosteric agonists (17, 24). In the case of α7 nAChRs, there is evidence for PAMs and allosteric agonists binding to an intrasubunit cavity located within the transmembrane domain (17, 25, 26).

**FIGURE 1.**
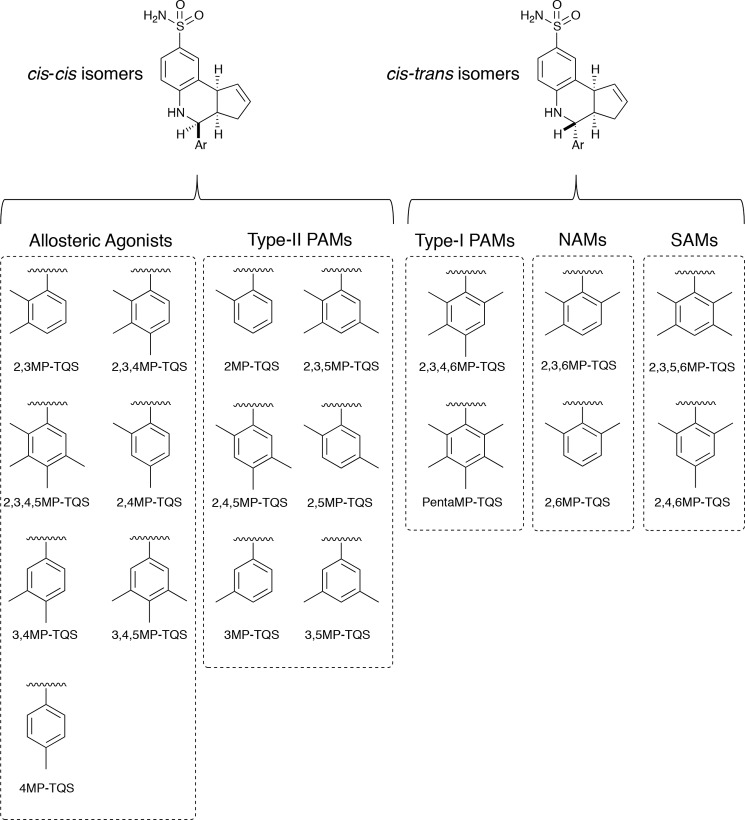
**Chemical structures of α7 nAChR allosteric modulators.** On the basis of experimental data obtained in the present study, compounds have been classified as allosteric agonists, desensitizing (type I) PAMs, nondesensitizing (type II) PAMs, NAMs, or SAMs. Compounds differ only in the pattern of methyl substitution at a single aromatic ring (designated *Ar* in the figure). Information concerning the ratio of *cis-cis*- and *cis-trans*-diastereoisomers obtained during synthesis is provided in the supplemental materials.

We have shown previously that minor changes in the chemical structure of nAChR allosteric modulators can result in dramatic differences in pharmacological properties (17, 24). For example, replacing a fluorine atom with a chlorine atom converts an α7-selective PAM into an allosteric agonist (24). The present study extends these findings, with the aim of identifying the influence of changes in chemical structure on the pharmacological properties of nAChR allosteric modulators. A series of compounds, differing only in methyl substitution of a single aromatic ring, have been examined (see Fig. 1). This series includes an α7-selective allosteric agonist, 4MP-TQS, a compound that has been examined previously (24), together with all other possible combinations of methyl substitutions at a single phenyl ring (18 additional compounds). The influence of changes in chemical structure has been examined on α7 nAChRs by means of two-electrode voltage-clamp recording and radioligand binding. Whereas previous studies of compounds with close chemical similarity to TQS have identified only allosteric agonists or type II PAMs (15, 17, 24), studies conducted with this series of 19 methyl-substituted compounds have revealed five distinct pharmacological effects on α7 nAChRs. In summary, the 19 methyl-substituted compounds examined in this study can be classified in one of five categories: allosteric agonists, type I PAMs, type II PAMs, negative allosteric modulators (NAMs), or silent allosteric modulators (SAMs).

## EXPERIMENTAL PROCEDURES

### 

#### 

##### Chemical Synthesis

Nineteen compounds were synthesized that differed only in the pattern of methyl substitution of an aromatic substituent located at position 2 of a tetrahydroisoquinoline ring (see Fig. 1). Compounds were prepared by InCl_3_-catalyzed reaction of sulfanilamide, cyclopentadiene, and the corresponding substituted benzaldehyde, according to methods described previously (27). The substituted benzaldehydes were either purchased commercially or prepared according to literature procedures (28). In most cases (13 of the 19 compounds), the *cis-cis*-diastereoisomer was obtained as the major product from the InCl_3_-catalyzed multicomponent reaction (see Fig. 1). However, in some cases (6 of the 19 compounds), the *cis-trans*-diastereoisomer was obtained as the major product (see Fig. 1). For all *cis-trans*-isomers and for two of the *cis-cis*-isomers, only a single diastereoisomer was detected by ^1^H NMR. Further details concerning the synthesis of these compounds and the major diastereoisomer obtained for each compound, together with the ratio of isomers present in the purified sample, are provided in the supplemental materials.

##### Subunit cDNAs and Plasmid Expression Vectors

Several plasmid constructs used in this study have been described previously. These constructs include plasmids containing human wild-type and mutant (M253L) nAChR α7 subunit cDNA constructs in plasmid pSP64GL (17, 29), human α7 nAChR in plasmid pcDNA3 (30), mouse 5-HT_3A_ in plasmid pRK5 (31), and *Caenorhabditis elegans* RIC-3 in plasmid pRK5 (32).

##### Xenopus laevis Oocyte Electrophysiology

Oocytes were isolated from female *X. laevis* and defolliculated as described previously (33). Heterologous expression was achieved by injection of either cRNA (6–12 ng) into oocyte cytoplasm in the case of wild-type and mutated α7 or plasmid cDNA constructs (10–30 ng) into oocyte nuclei in the case of 5-HT_3A_. *In vitro* transcription of cRNA was carried out using mMESSAGE mMACHINE SP6 transcription kit (Ambion, Huntington, UK). Oocytes were injected in a volume of 32.2 nl using a Drummond variable volume microinjector. Two electrode voltage-clamp recordings were performed (with the oocyte membrane potential held at −60 mV), as described previously (33) using a Warner Instruments OC-725C amplifier (Harvard Apparatus, Edenbridge, UK), PowerLab 8SP, and Chart 5 software (AD Instruments, Oxford, UK). Methyl-TQS compounds were dissolved in DMSO to generate 100 mm stock solutions. Compounds were applied to oocytes using a BPS-8 solenoid valve solution exchange system (ALA Scientific Inc., Westbury, NY), controlled by Chart software. For multiple comparisons of agonist activation rates, statistical significance was determined with a one-way analysis of variance (ANOVA). Statistical significance of desensitization rates was determined by paired Student's *t* tests. A *p* value of <0.05 was considered significant. The activation and desensitization phases of current responses were best fitted by a single exponential function.

##### Cell Culture

Human kidney tsA201 cells were cultured in Dulbecco's modified Eagle's medium (Invitrogen) containing 10% fetal calf serum (Sigma-Aldrich), penicillin (100 units/ml), and streptomycin (100 μg/ml) (Invitrogen). Cells were maintained in a humidified incubator containing 5% CO_2_ at 37 °C. Cells were co-transfected with human α7 nAChR cDNA and *C. elegans* RIC-3 cDNA using Effectene reagent (Qiagen) according to the manufacturer's instructions. After overnight incubation in Effectene, cells were incubated at 37 °C for 24–48 h before being assayed for radioligand binding.

##### Radioligand Binding

Radioligand binding to transiently transfected tsA201 cells was performed as described previously (30, 34) with [^3^H]α-bungarotoxin (specific activity, 56 Ci/mmol; Tocris Bioscience). Transfected cells were resuspended in Hank's buffered saline solution (Invitrogen) containing 1% bovine serum albumin and incubated with [^3^H]α-bungarotoxin for 2 h at 22 °C in a total volume of 150 μl. Nonspecific binding was determined in the presence of methyllycaconitine (MLA) (1 μm). Competition binding experiments were performed by incubating triplicate samples of transfected cells with [^3^H]α-bungarotoxin (10 nm), together with a range of concentrations (1–100 μm) of 2,3,6MP-TQS or 2,6MP-TQS and 100 μm of all other allosteric modulators in this study. Radioligand binding was assayed by filtration onto Whatman GF/A filters (presoaked in 0.5% polyethylenimine), followed by rapid washing with phosphate-buffered saline (Oxoid) using a Brandel cell harvester. Bound radioligand was determined by scintillation counting.

## RESULTS

Nineteen compounds were synthesized that share close chemical similarity to one another but form a series containing all possible combinations of methyl substitution on a single aromatic ring (Fig. 1). The majority of these compounds were obtained as the *cis-cis*-diastereoisomer but with varying degrees of diastereoselectivity (Fig. 1). However, those compounds containing an aromatic ring bearing two *ortho* methyl groups were obtained as the *cis-trans*-isomer with very high selectivity (Fig. 1). The pharmacological properties of all 19 compounds were examined on human α7 nAChRs expressed in *Xenopus* oocytes.

### 

#### 

##### Allosteric Agonist Activation of α7 nAChRs

**FIGURE 2.**
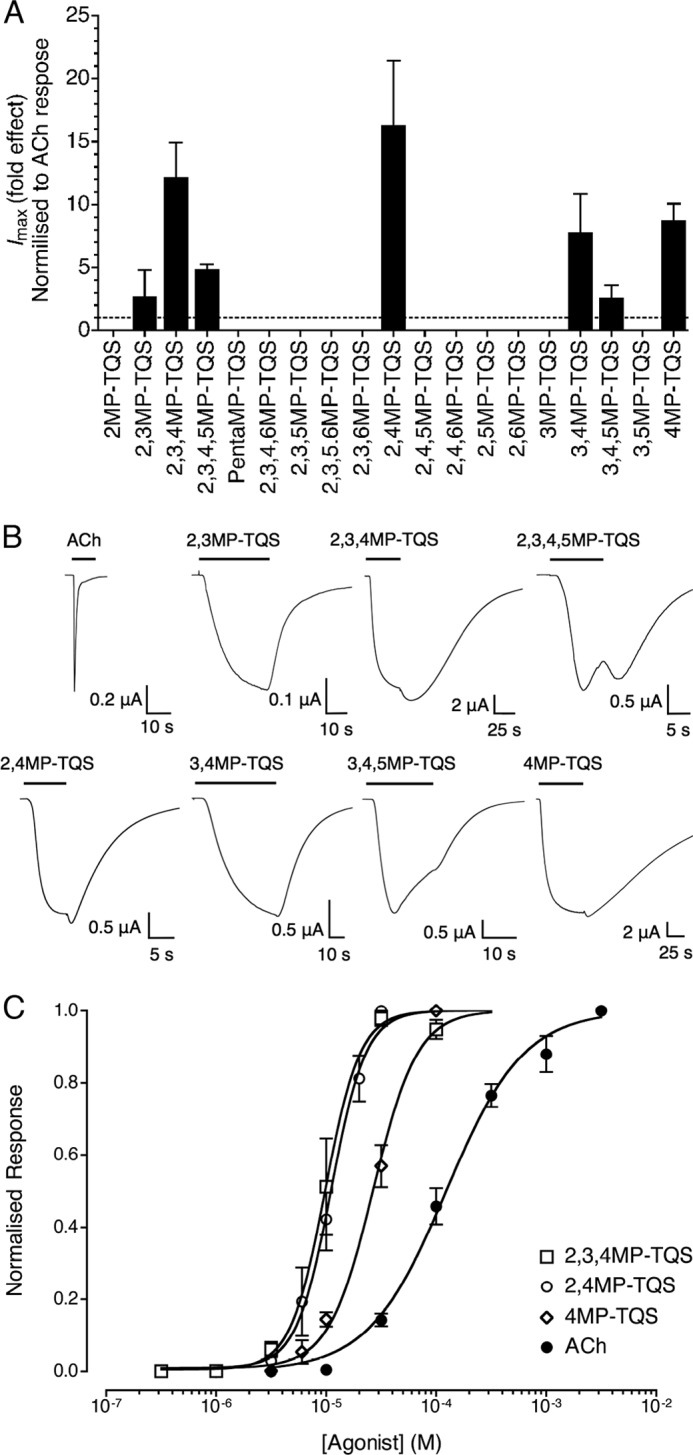
**Pharmacological properties of allosteric agonists on α7 nAChRs expressed in *X. laevis* oocytes.** *A*, bar graph illustrating agonist responses observed with 100 μm of each compound. Responses are normalized to the average response obtained upon application of acetylcholine alone at a maximum effective concentration (3 mm). Data are means ± S.E. of 3–15 independent experiments (Table 1). *B*, representative recordings are shown illustrating responses to the application of acetylcholine (3 mm) and of the allosteric agonists (100 μm). The *horizontal lines* indicate the duration of agonist applications. Responses have been normalized to their peak response. *C*, concentration-response data are presented for a range of concentrations of 2,3,4MP-TQS (*open squares*), 2,4MP-TQS (*open circles*), 4MP-TQS (*open diamonds*), and acetylcholine (*closed circles*). Data are means ± S.E. of at least three independent experiments, each from different oocytes. Data are normalized to agonist-induced responses induced by the maximal effective concentration.

Of the 19 methyl-substituted compounds examined, seven of these (2,3MP-TQS, 2,3,4MP-TQS, 2,3,4,5MP-TQS, 2,4MP-TQS, 3,4MP-TQS, 3,4,5MP-TQS, and 4MP-TQS) were found to have properties typical of α7 nAChR allosteric agonists (Fig. 2). In contrast to the rapidly desensitizing responses observed with orthosteric agonists such as acetylcholine, all seven of these methyl-substituted compounds activated α7 nAChRs with very much reduced levels of desensitization (Fig. 2*B*). Activation by the methyl-substituted compounds resulted in responses that were similar to responses with allosteric agonists of α7 nAChRs examined previously (17, 24, 35). Responses had a slow onset, were slow to reach a plateau, and had very different kinetics to activation by acetylcholine, which causes rapid desensitization (Fig. 2*B*). Agonist activation rates were analyzed from the start of agonist application to the peak response (Fig. 2*A* and Table 1). The rate of activation by all of the allosteric agonists examined was significantly slower compared with activation by acetylcholine (*p* < 0.01; Table 1). In addition, two of the allosteric agonists (3,4MP-TQS and 4MP-TQS) had significantly slower activation rates than the other five allosteric agonists (*p* < 0.05; Table 1).

**TABLE 1:** **Pharmacological and kinetic properties of α7 nAChR agonists** Data are means of 3–15 independent experiments ± S.E. EC_50_ values of all compounds examined were significantly different from that of acetylcholine (ANOVA and Dunnett's post hoc multiple comparison test; *p* < 0.01). For all compounds examined, Hill coefficients (*n*_H_) were significantly different from that of acetylcholine (ANOVA and Dunnett's post hoc multiple comparison test; *p* < 0.05). Activation rates correspond to the time constant for activation (τ) in the continuous presence of the agonist (ANOVA and Tukey's post-hoc test: *a* versus *b/c*, *p* < 0.01, *b* versus *c*, *p* < 0.05). Maximum current responses (*I*_max_) are presented as fold effects in which responses to allosteric agonists have been normalized to the mean response to a maximal concentration of acetylcholine (3 mm) obtained from the same oocyte. ND, not determined.

Agonist	EC_50_	*n*_H_	Activation rate τ	*I*_max_
	μ*m*		*s*	*Fold effect*
Acetylcholine	128 ± 12	1.3 ± 0.2	0.22 ± 0.11*^a^*	1
2,3MP-TQS	ND	ND	14 ± 2.4*^b^*	2.7 ± 2.1
2,3,4MP-TQS	9.8 ± 0.8	2.7 ± 0.9	9.4 ± 0.9*^b^*	12 ± 2.7
2,3,4,5MP-TQS	ND	ND	6.4 ± 1.0*^b^*	4.9 ± 0.4
2,4MP-TQS	11 ± 1.0	2.8 ± 0.6	7.5 ± 0.4*^b^*	16 ± 5.1
3,4MP-TQS	ND	ND	29 ± 4.2*^c^*	7.8 ± 3.0
3,4,5MP-TQS	ND	ND	7.2 ± 0.6*^b^*	2.6 ± 1.0
4MP-TQS	27 ± 3.1	2.2 ± 0.2	25 ± 2.4*^c^*	8.7 ± 1.3

Concentration-response curves were generated for three of the seven allosteric agonists (2,3,4MP-TQS, 2,4MP-TQS, and 4MP-TQS; Fig. 2*C*). As was observed with α7-selective allosteric agonists examined previously (17, 24, 35), concentrations of 2,3,4MP-TQS, 2,4MP-TQS, and 4MP-TQS that caused half-maximal activation of α7 nAChR were significantly lower than that of the endogenous orthosteric agonist, acetylcholine (Table 1). In addition, concentration-response curves for 2,3,4MP-TQS, 2,4MP-TQS, and 4MP-TQS were significantly steeper than for acetylcholine (Table 1).

##### Type I and Type II Positive Allosteric Modulation of α7 nAChRs

**FIGURE 3.**
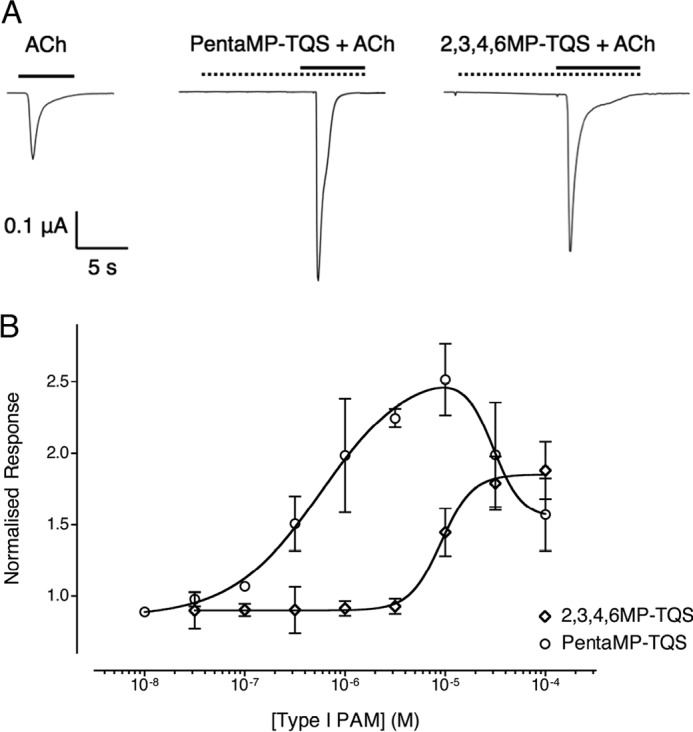
**Pharmacological properties of type I PAMs on α7 nAChRs expressed in *X. laevis* oocytes.** *A*, representative recordings are shown illustrating responses to the application of acetylcholine (100 μm) alone or to a maximal concentration of the PAM (PentaMP-TQS 10 μm; 2,3,4,6MP-TQS 100 μm) preapplied for 10 s and then co-applied with acetylcholine (100 μm). *Solid horizontal lines* indicate the application of acetylcholine, and *dotted horizontal lines* indicate the application of the PAM. *B*, concentration-response data are presented for a range of concentrations of 2,3,4,6MP-TQS (*open diamonds*) and PentaMP-TQS (*open circles*) on responses evoked by a submaximal (EC_50_) concentration of acetylcholine with wild-type α7 nAChRs. The PAM was preapplied for 10 s and then co-applied with acetylcholine (100 μm). Data are means ± S.E. of at least three independent experiments, each from different oocytes. Data are normalized to a submaximal (EC_50_) concentration of acetylcholine (100 μm).

**FIGURE 4.**
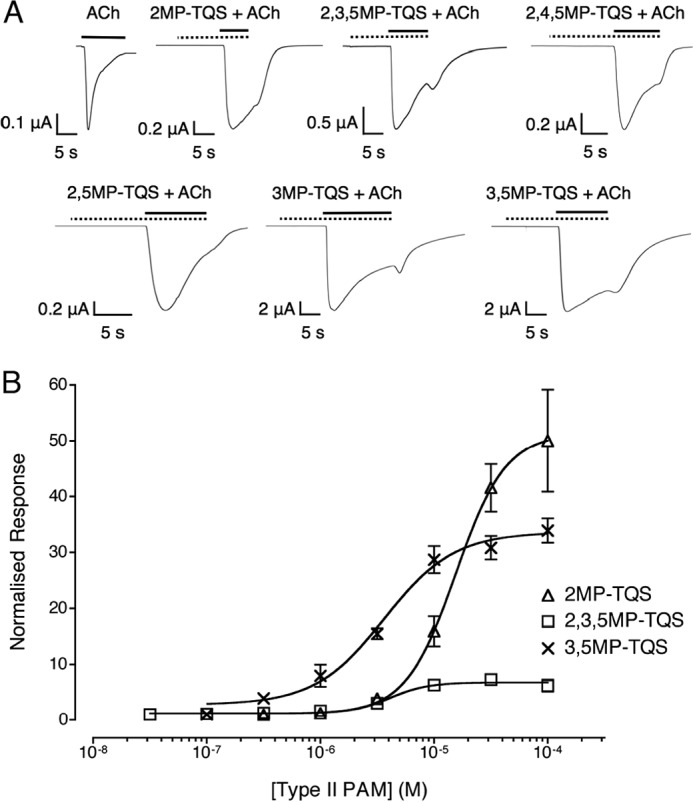
**Pharmacological properties of type II PAMs on α7 nAChRs expressed in *X. laevis* oocytes.** *A*, representative recordings are shown illustrating responses to the application of acetylcholine (100 μm) and a maximal concentration of the PAM (100 μm) preapplied for 10 s and then co-applied with acetylcholine (100 μm). *Solid horizontal lines* indicate the application of acetylcholine, and *dotted horizontal lines* indicate the application of the PAM. Responses have been normalized to their peak response. *B*, concentration-response data are presented for a range of concentrations of 2MP-TQS (*open triangles*), 2,3,5MP-TQS (*open squares*), and 3,5MP-TQS (*crosses*) on responses evoked by a submaximal (EC_50_) concentration of acetylcholine (100 μm) with wild-type α7 nAChRs. The PAM was preapplied for 10 s and then co-applied with acetylcholine (100 μm). Data are means ± S.E. of at least three independent experiments, each from different oocytes. Data are normalized to a submaximal (EC_50_) concentration of acetylcholine (100 μm).

For those compounds that did not have allosteric agonist activity, their ability to potentiate or inhibit acetylcholine-induced responses was examined. Potentiation of acetylcholine-evoked responses was observed with eight of the twelve compounds that lacked agonist activity (2MP-TQS, 2,3,4,6MP-TQS, 2,3,5MP-TQS, 2,4,5MP-TQS, 2,5MP-TQS, 3MP-TQS, 3,5MP-TQS, and PentaMP-TQS; Figs. 3*A* and 4*A* and Table 2). The lack of intrinsic agonist activity of these compounds, together with their ability to potentiate acetylcholine-induced responses is characteristic of a group of compounds that have been described as PAMs (20).

**TABLE 2:** **Pharmacological and kinetic properties of α7 nAChRs** Data are means of 3–28 independent experiments ± S.E. Fold potentiation refers to the increase in current, normalized to the mean response to a submaximal concentration of acetylcholine (100 μm) obtained from the same oocyte. Desensitization rates correspond to the time constant for desensitization (τ) in the continuous presence of acetylcholine and, when appropriate, the PAM (significant differences to desensitization rates with acetylcholine alone were determined by pairwise Student's *t* tests. NA, not applicable; ND, not determined.

Ligand	EC_50_	Fold potentiation	Desensitization rate τ	PAM type	
				I	II
	μ*m*		*s*		
Acetylcholine	128 ± 12	NA	1.5 ± 0.1		
2MP-TQS	16 ± 1.3	39 ± 7.5	16 ± 3.2*^a^*		×
PentaMP-TQS	0.55 ± 0.36	2.4 ± 0.3	3.5 ± 1.3	×	
2,3,4,6MP-TQS	9.1 ± 2.0	2.3 ± 0.3	1.1 ± 0.1	×	
2,3,5MP-TQS	3.9 ± 0.7	6.4 ± 0.5	7.6 ± 1.0*^b^*		×
2,4,5MP-TQS	ND	11 ± 2.7	4.0 ± 0.2*^b^*		×
2,5MP-TQS	ND	1.8 ± 0.4	6.1 ± 0.8*^b^*		×
3MP-TQS	ND	22 ± 2.1	33 ± 9.9*^a^*		×
3,5MP-TQS	3.7 ± 1.2	33 ± 4.9	16 ± 0.6*^c^*		×

*^a^ p* < 0.05.

*^b^ p* < 0.01.

*^c^ p* < 0.001.

None of the PAMs examined had a significant influence on the rate of receptor activation compared with acetylcholine alone. Although the activation rates did not differ, differences were observed in the rate of desensitization of α7 nAChRs with these PAMs, as has been described previously with other PAMs (24). Two of the compounds (2,3,4,6MP-TQS and PentaMP-TQS) displayed properties characteristic of type I PAMs, having no significant difference on the rate of receptor desensitization compared with acetylcholine (*p* > 0.05; Fig. 3*A* and Table 2). The other six compounds (2MP-TQS, 2,3,5MP-TQS, 2,4,5MP-TQS, 2,5MP-TQS, 3MP-TQS, and 3,5MP-TQS) caused significant slowing in the rate of receptor desensitization compared with acetylcholine (*p* < 0.05; Fig. 4*A* and Table 2), a property that is characteristic of type II PAMs.

Concentration-response curves were generated with the two type I PAMs (2,3,4,6MP-TQS and PentaMP-TQS; Fig. 3*B*) and with three of the six type II PAMs (2MP-TQS, 2,3,5MP-TQS and 3,5MP-TQS; Fig. 4*B*). With PentaMP-TQS, a bell-shaped concentration-response curve was observed (Fig. 4*B*). The potentiating component of the concentration-response curve with PentaMP-TQS produced a half-maximal potentiation at a concentration of 0.55 ± 0.36 μm, although the true potency is likely to be obscured by the inhibitory component of the concentration-response curve. A maximal potentiation of 2.4 ± 0.3-fold of the control acetylcholine response was observed at ∼10 μm PentaMP-TQS (Fig. 3*B*). Concentration-response experiments were also performed with 2,3,4,6MP-TQS (Fig. 3*B*), and a half-maximal potentiation was observed at a concentration of 9.1 ± 2.0 μm. Concentration-dependent potentiation of acetylcholine responses was seen for all three type II PAMs examined (2MP-TQS, 2,3,5MP-TQS, and 3,5MP-TQS; Fig. 4*B* and Table 2), with levels of potentiation of up to 39 ± 7.5-fold (Table 2).

##### Negative Allosteric Modulation of α7 nAChRs

**FIGURE 5.**
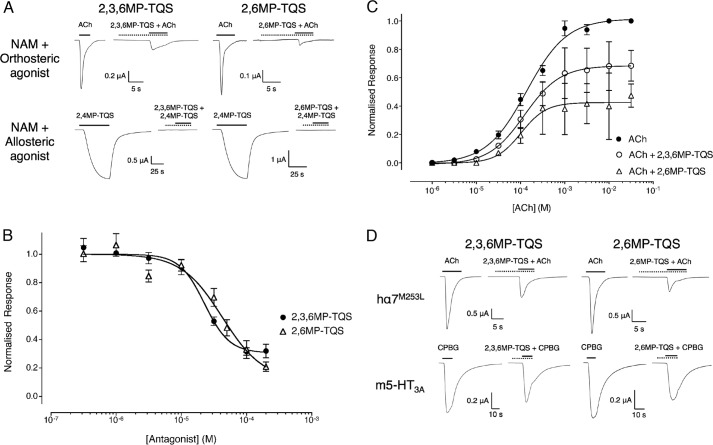
**Pharmacological properties of antagonists (NAMs) on human α7 nAChRs and mouse 5HT_3A_Rs expressed in *X. laevis* oocytes.** *A*, representative recordings are shown illustrating responses with wild-type α7. In the *top panel*, *Orthosteric agonist* indicates the application of acetylcholine (100 μm) and a maximal concentration of the antagonist (200 μm) preapplied for 10 s and then co-applied with acetylcholine (100 μm). In the *bottom panel*, *Allosteric agonist* indicates the application of 2,4MP-TQS (10 μm) and a maximal concentration of the antagonist (200 μm) preapplied for 10 s and then co-applied with 2,4MP-TQS (10 μm). *Solid horizontal lines* indicate the application of the agonist, and *dotted horizontal lines* indicate the application of the NAM. *B*, concentration-response data are presented illustrating the ability of 2,3,6MP-TQS (*filled circles*) and 2,6MP-TQS (*open triangles*) to inhibit responses evoked by a submaximal (EC_50_) concentration of acetylcholine (100 μm) on wild-type α7. The antagonist was preapplied for 10 s and then co-applied with acetylcholine (100 μm). Data are means ± S.E. of at least three independent experiments, each from different oocytes. Data are normalized to a submaximal (EC_50_) concentration of acetylcholine (100 μm). *C*, both 2,3,6MP-TQS and 2,6MP-TQS are noncompetitive antagonists of acetylcholine. Concentration-response data are presented for a range of concentrations of acetylcholine acting on wild-type α7 nAChRs in either the absence (*filled circles*) or presence of 20 μm 2,3,6MP-TQS (*open circles*) or 40 μm 2,6MP-TQS (*open triangles*). In all cases the antagonist was preapplied for 10 s and then co-applied with acetylcholine. Data are means ± S.E. of at least three independent experiments, each from different oocytes. Data are normalized to acetylcholine (3 mm). *D*, in the *top panels*, representative recordings are shown illustrating responses on α7^M253L^ to the application of acetylcholine (100 μm) (*left panel*) and a maximal concentration of the antagonist (200 μm) preapplied for 10 s and then co-applied with acetylcholine (100 μm) (*right panel*). In the *bottom panels*, the same protocol was used for the 5-HT_3A_ receptors except CPBG (1 μm) was used as the agonist instead of acetylcholine. *Solid horizontal lines* indicate the application of CPBG, and *dotted horizontal lines* indicate the application of the PAM.

Two of the methyl-substituted compounds (2,3,6MP-TQS and 2,6MP-TQS) caused inhibition of responses evoked by acetylcholine on human α7 nAChRs (Fig. 5, *A* and *B*). This inhibition was concentration-dependent (*IC*_50_ = 23 ± 2.8 and 43 ± 14 μm for 2,3,6MP-TQS and 2,6MP-TQS, respectively). The maximum inhibition of an EC_50_ concentration of acetylcholine (100 μm) that was observed was 68 ± 2.8% with 2,3,6MP-TQS and 74 ± 4.2% with 2,6MP-TQS (Fig. 5*B*). In addition to their antagonist effects on acetylcholine, 2,3,6MP-TQS and 2,6MP-TQS both acted as antagonists when co-applied with allosteric agonists. For example, a complete block of the response to the allosteric agonist 2,4MP-TQS (applied at 10 μm) was observed when co-applied with 100 μm of either 2,3,6MP-TQS or 2,6MP-TQS (Fig. 5*A*). A plausible explanation for these effects might be that 2,3,6MP-TQS and 2,6MP-TQS were acting as noncompetitive antagonists with respect to acetylcholine but binding at the same site as the allosteric agonists. In contrast to the effects of MLA as a competitive antagonist of acetylcholine (17), antagonism by 2,3,6MP-TQS and 2,6MP-TQS was not surmountable (Fig. 5*C*) and had no significant effect on the EC_50_ value compared with acetylcholine alone (EC_50_ = 120 ± 32 and 104 ± 27 μm for 2,3,6MP-TQS and 2,6MP-TQS, respectively). This feature is characteristic of noncompetitive antagonism and supports the conclusion that 2,3,6MP-TQS and 2,6MP-TQS bind at a site that is distinct from the conventional orthosteric binding site.

##### Silent Allosteric Modulators of α7 nAChRs

**FIGURE 6.**
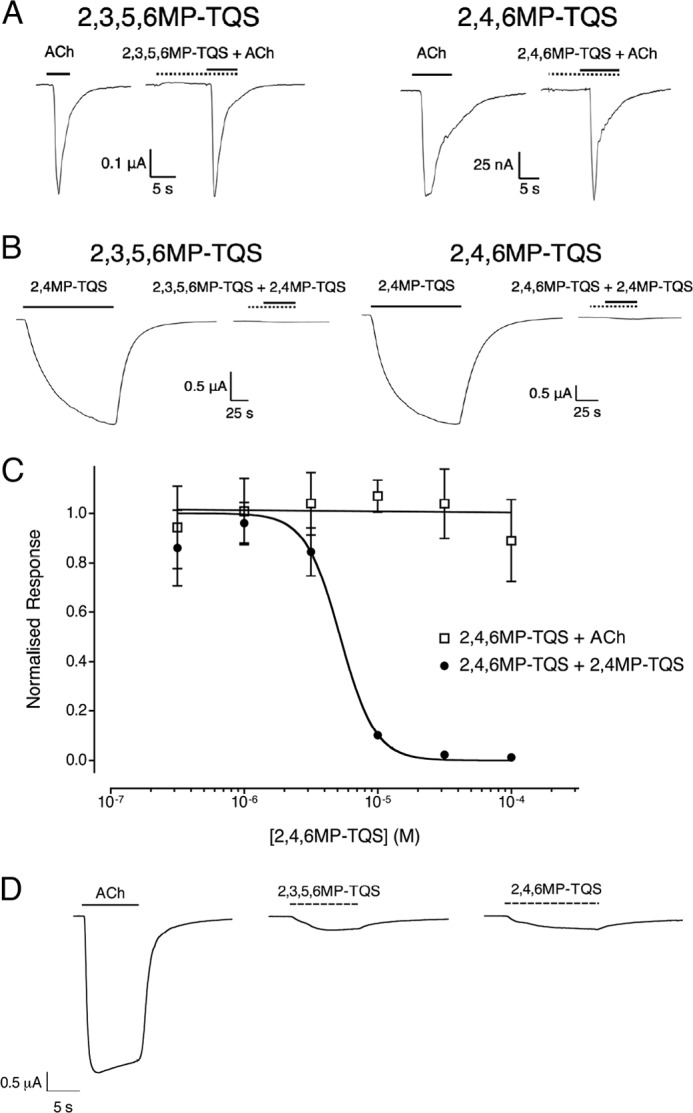
**Pharmacological properties of SAMs on α7 nAChRs expressed in *X. laevis* oocytes.** *A*, representative recordings are shown illustrating responses to the application of acetylcholine (100 μm) (*left panel*) and a maximal concentration of the SAM (100 μm) preapplied for 10 s and then co-applied with acetylcholine (100 μm) (*right panel*). *B*, representative recordings are also shown illustrating responses to the application of 2,4MP-TQS (10 μm) (*left panel*) and a maximal concentration of the SAM (100 μm) preapplied for 10 s and then co-applied with 2,4MP-TQS (100 μm) (*right panel*). *Solid horizontal lines* indicate the application of acetylcholine, and *dotted horizontal lines* indicate the application of the SAM. *C*, concentration-response data are presented illustrating the ability of 2,4,6MP-TQS to have no effect on a submaximal (EC_50_) concentration of acetylcholine (100 μm) (□) but to inhibit responses evoked by a submaximal (EC_50_) concentration of 2,4MP-TQS (10 μm) (●). The compound was preapplied for 10 s and then co-applied with either agonist. Data are means ± S.E. of at least three independent experiments, each from different oocytes. Data are normalized to a submaximal (EC_50_) concentration of either acetylcholine (100 μm) or 2,4MP-TQS (10 μm). *D*, the α7 nAChR transmembrane mutation L247T converts SAMs (2,3,5,6MP-TQS and 2,4,6MP-TQS) into agonists. Representative recordings are shown illustrating responses to the application of acetylcholine (10 μm) (*left panel*), 2,3,5,6MP-TQS (10 μm) (*middle panel*), and 2,4,6MP-TQS (10 μm) (*right panel*) on α7^L247T^ nAChRs. *Solid horizontal lines* indicate the application of acetylcholine, and *dotted horizontal lines* indicate the application of the SAM.

The remaining two compounds in the series of 19 methyl-substituted compounds (2,3,5,6MP-TQS and 2,4,6MP-TQS) displayed no obvious pharmacological effects on α7 nAChRs, either when applied alone (*i.e.* no evidence of allosteric agonist activation; Fig. 6*A*) or when co-applied with acetylcholine (*i.e.* no PAM or antagonist activity; Fig. 6*A*). However, when either of these two compounds was co-applied with an allosteric agonist (2,4MP-TQS), both caused an inhibition of allosteric agonist activity (Fig. 6*B*). Co-application of 2,4,6MP-TQS resulted in a concentration-dependent inhibition of responses evoked by 2,4MP-TQS (*IC*_50_ = 5.2 ± 0.8 μm; Fig. 6*C*). Co-application of 2,3,5,6MP-TQS (100 μm) with the allosteric agonist, 2,4MP-TQS (10 μm) also resulted in a full block of the agonist response with 2,4MP-TQS (Fig. 6*B*). A plausible explanation of these effects would be that 2,3,5,6MP-TQS and 2,4,6MP-TQS are binding at the same site as the allosteric agonists and causing inhibition by displacement of the allosteric agonist from its transmembrane binding site.

##### Influence of the Transmembrane L247T Mutation

Mutations of amino acid Leu-247 (such as L247T) in α7 nAChRs have been found to have dramatic effects on the functional properties of the receptor (36). The L247T mutation causes some competitive antagonists to act as agonists (37), and it also causes some PAMs, such as TQS, to act as agonists (17). The influence of the L247T mutation was examined on those compounds that displayed no agonist activity and neither potentiated nor inhibited acetylcholine-evoked responses (2,3,5,6MP-TQS and 2,4,6MP-TQS). Interestingly, it was found that the L247T mutation converts both 2,3,5,6MP-TQS and 2,4,6MP-TQS, which are silent allosteric modulators on wild-type α7 nAChRs, into weak nondesensitizing agonists, a property that is characteristic of allosteric agonists acting on α7 nAChRs (Fig. 6*D*). This supports the conclusion that 2,3,5,6MP-TQS and 2,4,6MP-TQS are binding at the previously identified transmembrane allosteric site (17, 25) and should be classified as SAMs.

##### Influence of the Transmembrane M253L Mutation

Recent studies have proposed that α7-selective PAMs and allosteric agonists can act via a transmembrane binding site (17, 25). One of the lines of evidence supporting this proposal is that potentiation by TQS and also activation by 4BP-TQS is not observed with α7 receptors containing the transmembrane mutation M253L (17). In contrast, M253L has been shown to have no significant effect on activation by the conventional orthosteric agonist acetylcholine (17, 25).

The effect of the M253L mutation on activation by the six allosteric agonists (2,3MP-TQS, 2,3,4MP-TQS, 2,3,4,5MP-TQS, 2,4MP-TQS, 3,4MP-TQS, and 3,4,5MP-TQS) was examined. In all cases, the M253L mutation caused complete loss of agonist activation. In addition, the effect of the M253L mutation on allosteric potentiation by the eight PAMs (2MP-TQS, 2,3,4,6MP-TQS, 2,3,5MP-TQS, 2,4,5MP-TQS, 2,5MP-TQS, 3MP-TQS, 3,5MP-TQS, and PentaMP-TQS) was examined. As has been reported previously for potentiation by TQS (17), the M253L mutation caused a complete loss of PAM activity with all of these compounds. The simplest explanation for these findings may be that all of these compounds (allosteric agonists and PAMs in the series of methyl-substituted compounds) act via a shared allosteric binding site, as has been proposed previously for TQS (a type II PAM) and 4BP-TQS (an allosteric agonist) (17).

The effect of the M253L mutation was also examined on those compounds that act as antagonists of acetylcholine (2,3,6MP-TQS and 2,6MP-TQS) (Fig. 5*D*). Responses evoked by an EC_50_ concentration of acetylcholine (100 μm) were inhibited by 64 ± 4.0% with 2,3,6MP-TQS and by 73 ± 1.7% with 2,6MP-TQS. These levels of inhibition are not significantly different to that observed with wild-type α7 nAChRs (Student's *t* test, *p* > 0.05). We note, however, that the M253L mutation was generated so as to introduce into the nAChR α7 subunit the amino acid occurring at the analogous position in the mouse 5-HT3A subunit (17, 25). For this reason, the effect of 2,3,6MP-TQS and 2,6MP-TQS was examined on the mouse 5-HT3A subunit expressed in oocytes. A maximal concentration of either 2,3,6MP-TQS or 2,6MP-TQS was preapplied and then co-applied with the 5-HT_3_ receptor (5-HT_3_R) agonist 1-(3-chlorophenyl)biguanide hydrochloride (CPBG) (1 μm). Both TQS compounds resulted in inhibition of responses evoked by CPBG (Fig. 5*D*). The finding that 2,3,6MP-TQS and 2,6MP-TQS act as inhibitors of both α7 nAChRs and 5-HT_3_R may explain why the M253L mutation in α7 has no significant effect on allosteric modulation by 2,3,6MP-TQS or 2,6MP-TQS but has a dramatic effect on modulation by other chemically similar allosteric modulators.

##### Radioligand Binding

**FIGURE 7.**
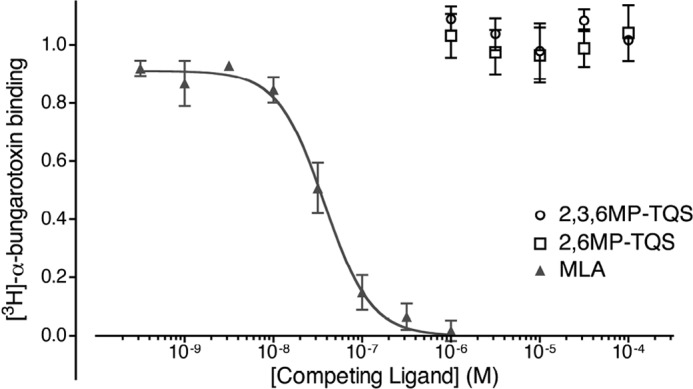
**Competition radioligand binding on tsA201 cells transiently co-transfected with human α7 nAChR cDNA and *C. elegans* RIC-3 cDNA.** Equilibrium radioligand binding was performed with [^3^H]α-bungarotoxin (10 nm) in the presence of varying concentrations of competing ligands (MLA or allosteric modulators). No significant displacement of [^3^H]α-bungarotoxin was observed with any of the 19 methyl-substituted compounds up to the maximum texted (100 μm). (Note that for simplicity, data are shown only for two allosteric modulators (2,3,6MP-TQS and 2,6MP-TQS).) In contrast to the findings with allosteric modulators, complete displacement of [^3^H]α-bungarotoxin was observed with the orthosteric ligand MLA. Data are means of triplicate samples from a single experiment, from three independent experiments ± S.E. Data are normalized to the maximal [^3^H]α-bungarotoxin binding.

The dramatic effect of the M253L mutation on those methyl-substituted compounds displaying agonist or PAM activity helps to provide support for the conclusion that these compounds may act at a previously proposed transmembrane allosteric site (17, 25). In contrast, the common inhibitory effect of 2,3,6MP-TQS and 2,6MP-TQS on α7 nAChRs and on 5-HT_3A_ receptors means that no conclusions can be made from studies with the M253L mutation concerning the two methyl-substituted compounds that display antagonist effects on responses evoked by acetylcholine. Our working hypothesis is that all of the structurally similar methyl-substituted compounds examined in this study are likely to interact with a broadly similar allosteric site on the α7 nAChR, as discussed previously for other related compounds (17, 24). A consequence of this hypothesis would be that the antagonism of acetylcholine-evoked responses that has been observed with 2,3,6MP-TQS and 2,6MP-TQS is due to a noncompetitive mechanism of action. With the aim of testing this hypothesis, we examined the ability of methyl-substituted compounds to displace the binding of an orthosteric radioligand ([^3^H]α-bungarotoxin). No significant displacement of [^3^H]α-bungarotoxin binding was observed with any of the 19 methyl-substituted compounds, even at the maximum concentration tested (100 μm; ANOVA; *p* = 1.0). Results obtained from competition binding studies with 2,3,6MP-TQS and 2,6MP-TQS are illustrated in Fig. 7. This suggests that none of the methyl-substituted compounds bind competitively at the orthosteric nicotinic ligand-binding site. In contrast, as has been reported previously (38), the competitive antagonist MLA causes complete displacement of [^3^H]α-bungarotoxin from α7 nAChRs (*IC*_50_ = 37 ± 4.8 nm; Fig. 7). This provides support for our conclusion that the antagonism of acetylcholine-evoked responses by 2,3,6MP-TQS and 2,6MP-TQS is by a noncompetitive (allosteric) mechanism and that all of the methyl-substituted compounds examined in this study act at a site distinct from the conventional orthosteric binding site. It is also consistent with our evidence that the antagonism of acetylcholine-evoked responses by 2,3,6MP-TQS and 2,6MP-TQS is not surmountable (Fig. 5*C*).

## DISCUSSION

In the present study, the pharmacological properties of 19 methyl-substituted compounds have been examined. Evidence has been obtained indicating that six of these compounds (2,3MP-TQS, 2,3,4MP-TQS, 2,3,4,5MP-TQS, 2,4MP-TQS, 3,4MP-TQS, and 3,4,5MP-TQS) act as allosteric agonists of α7 nAChRs. Previous studies (24, 25) have provided evidence that similar compounds (for example 4BP-TQS) interact with an intrasubunit transmembrane cavity in α7 nAChRs (17, 24). It seems plausible that the allosteric agonists examined in this study act via a similar site and by a similar mechanism of action.

Several of the compounds examined in the present study lack allosteric agonist activity but cause dramatic potentiation of responses evoked by acetylcholine (PAM activity). Based on their effect on the rate of agonist-evoked desensitization, compounds were identified that fulfill the criteria of both type I PAMs (which have no effect on receptor desensitization) and type II PAMs (which cause a slowing of desensitization were seen within the series). Two of the compounds examined (2,3,4,6MP-TQS and PentaMP-TQS) resembled type I PAMs, whereas six of the compounds (2MP-TQS, 2,3,5MP-TQS, 2,4,5MP-TQS, 2,5MP-TQS, 3MP-TQS, and 3,5MP-TQS) resembled type II PAMs. Compounds belonging to both groups of PAM were effective in shifting the potency of acetylcholine to the left and increasing the maximum efficacies, a phenomenon that is characteristic of nAChR PAMs (15, 39–41).

Work described here and elsewhere supports the conclusion that allosteric agonists and PAMs of α7 nAChRs can bind to a common transmembrane site (25, 26). This is supported by evidence that the effects of both allosteric agonists and of PAMs can be blocked completely by a single point mutation (M253L) located in the transmembrane region, a mutation that has no significant effect on agonist activation by the orthosteric agonist acetylcholine (17, 25).

Two of the compounds examined in this series (2,3,6MP-TQS and 2,6MP-TQS) lacked allosteric agonist activity but caused dramatic inhibition of responses evoked by acetylcholine and also of responses evoked by an allosteric agonist (2,4MP-TQS). When these compounds were examined on the α7^M253L^ nAChR, the mutation was found to have no significant effect on the level of inhibition by these compounds. However, the M253L mutation was generated so as to introduce into the nAChR α7 subunit the amino acid occurring at the analogous position in the mouse 5-HT3A subunit (17, 25). Our finding that responses evoked by the orthosteric agonist CPBG on 5-HT_3_Rs were inhibited by 2,3,6MP-TQS and 2,6MP-TQS may explain why the M253L mutation had no effect on the antagonist activity of these compounds on α7 nAChRs. This is because the M253L mutation was designed to change the existing methionine in α7 to the corresponding amino acid (leucine) at the analogous position in the mouse 5-HT3A subunit (25).

Evidence to support the conclusion that these antagonists bind noncompetitively with respect to acetylcholine is provided by data demonstrating that the antagonism by 2,3,6MP-TQS and 2,6MP-TQS is not surmountable (Fig. 5*C*). In addition, competition radioligand binding data provide further support for the conclusion that both 2,3,6MP-TQS and 2,6MP-TQS bind at a site other than the conventional orthosteric binding site. The structural similarity between 2,3,6MP-TQS and 2,6MP-TQS with that of other compounds differing only in methyl substitution of a phenyl ring (including allosteric agonists and PAMs) suggests that the most likely explanation for the inhibition of acetylcholine responses is that these compounds are binding noncompetitively with respect to acetylcholine and at a common or overlapping transmembrane site to that of the allosteric agonists and PAMs examined here.

As others have done previously (42, 43), we have used the term “silent allosteric modulator” (SAM) to denote compounds that interact with an allosteric site but that do not exert a modulatory effect on responses to the orthosteric agonist (*i.e.* neither a positive or negative allosteric effect) and that do have modulatory effects on compounds that interact at the same allosteric site. Two of the compounds examined in this study (2,3,5,6MP-TQS and 2,4,6MP-TQS) exhibited no allosteric agonist activity and neither potentiated nor inhibited responses evoked by acetylcholine. They did, however, act as antagonists of allosteric agonists. The simplest explanation for this observation is that 2,3,5,6MP-TQS and 2,4,6MP-TQS interact with the same allosteric site as allosteric agonists such as 4MP-TQS (17, 24) and can therefore be considered as being SAMs. The fact that neither compound caused displacement of [^3^H]α-bungarotoxin from its orthosteric binding site supports this conclusion. In this respect, they can be considered as acting in a manner that is analogous to previously described SAMs of G protein-coupled receptors (42, 43). Similarly, it has been shown previously that α7 nAChR PAMs such as TQS can act as antagonists of responses evoked by allosteric agonists such as 4BP-TQS (24, 35), presumably because they are binding competitively to a common site. The two compounds that acted as SAMs on wild-type α7 nAChRs (2,3,5,6MP-TQS and 2,4,6MP-TQS) both displayed agonist activity on mutant α7^L247T^ nAChRs. The L247T mutation is known to have a higher frequency of spontaneous openings (44), so it is possible that binding of 2,3,5,6MP-TQS or 2,4,6MP-TQS stabilizes the open conformation of the receptor, resulting in agonist activity.

Synthesis of the methyl-substituted compounds in the InCl_3_-catalyzed reaction can potentially result in of the formation of up to four different diastereoisomers. Typically, however, only two isomers are observed, classified as *cis-cis* or *cis-trans* on the basis of the relative stereochemistry of the three stereocenters on the isoquinoline ring (Fig. 1). The stereochemistry of the major isomer of each of the methyl-substituted compounds described in this study was assigned on the basis of the coupling constants observed in the ^1^H NMR (see supplemental materials for details). In most cases, the *cis-cis*-isomer was obtained as the major product, but reactions with more hindered benzaldehydes, containing two methyl groups *ortho* to the aldehyde group, gave the *cis-trans*-isomer as the major product (Fig. 1). It is likely that the presence of these two methyl groups leads the corresponding imine to adopt a very different conformation to avoid steric clash between the imine and the nearby methyl groups. This in turn is likely to raise the energy of the transition state, leading to the formation of the relatively hindered *cis-cis*-isomer during the cyclization reaction. As a consequence, the formation of the *cis-trans*-isomer becomes preferred to the extent that it is the sole product observed with these more hindered imines. Interestingly, all of the compounds that have been classified as allosteric agonists or as type II PAMs were found to be *cis-cis*-diastereoisomers, whereas all of the NAMs, SAMs, and type I PAMs were *cis-trans*-diastereoisomers.

Whereas we have previously obtained evidence that the size of the group attached to position 4 of the phenyl ring of TQS compounds can influence allosteric properties (24), our present findings indicate the role of groups attached at positions 2 and 6. These conclusions about the importance of positions 2 and 6 are also consistent with data obtained from all of the compounds similar to TQS that we have examined previously, all of which are either allosteric agonists or type II PAMs (17). All of these compound are *cis-cis*-diastereoisomers, and none contain substituents at both positions 2 and 6 (17). Our findings are also consistent with recent studies demonstrating that the *cis-cis*-(+)-enantiomer of 4BP-TQS (GAT107) is active as an allosteric agonist (45, 46).

In conclusion, we have synthesized a series of 19 compounds, containing all possible variations of methyl substitution at a single aromatic ring. Whereas previous studies of compounds with close chemical similarity to TQS have identified only allosteric agonists or type II PAMs (15, 17, 24), studies conducted with this series of 19 methyl-substituted compounds have revealed five distinct pharmacological effects on α7 nAChRs. In addition to allosteric agonists and type II PAMs, we have identified type I PAMs and also compounds that reduce agonist-evoked responses and can be considered as NAMs (are, alternatively, as noncompetitive antagonists). Finally, compounds have been identified that have no significant effect on orthosteric agonist-evoked responses but block responses to allosteric agonists (classified as SAMs). In summary, the 19 methyl-substituted compounds examined in this study (Fig. 1) can be classified in one of five categories: allosteric agonists (7 of the 19 compounds), type I PAMs (2 compounds), type II PAMs (6 compounds), NAMs (2 compounds), or SAMs (2 compounds). The data we have obtained are consistent with all of these compounds interacting with a common transmembrane allosteric site, as has been proposed previously for other allosteric modulators of α7 nAChRs (17, 24–26).

## Footnotes

5 The abbreviations used are:

nAChR: nicotinic acetylcholine receptor
2MP-TQS: *cis-cis*-4-*o*-tolyl-3a,4,5,9b-tetrahydro-3*H*-cyclopenta[c]quinoline-8-sulfonamide
2,3MP-TQS: *cis-cis*-4-(2,3-dimethylphenyl)-3a,4,5,9b-tetrahydro-3*H*-cyclopenta[c]quinoline-8-sulfonamide
2,3,4MP-TQS: *cis-cis*-4-(2,3,4-trimethylphenyl)-3a,4,5,9b-tetrahydro-3H-cyclopenta[c]quinoline-8-sulfonamide
2,3,4,5MP-TQS: *cis-cis*-4-(2,3,4,5-Tetramethylphenyl)-3a,4,5,9b-tetrahydro-3H-cyclopenta[c]quinoline-8-sulfonamide
2,3,4,6MP-TQS: *cis-trans*-4-(2,3,4,6-tetramethylphenyl)-3a,4,5,9b-tetrahydro-3H-cyclopenta[c]quinoline-8-sulfonamide
2,3,5MP-TQS: *cis-cis*-4-(2,3,5-trimethylphenyl)-3a,4,5,9b-tetrahydro-3H-cyclopenta[c]quinoline-8-sulfonamide
2,3,5,6MP-TQS: *cis-trans*-4-(2,3,5,6-tetramethylphenyl)-3a,4,5,9b-tetrahydro-3*H*-cyclopenta[c]quinoline-8-sulfonamide
2,3,6MP-TQS: *cis-trans*-4-(2,3,6-trimethylphenyl)-3a,4,5,9b-tetrahydro-3H-cyclopenta[c]quinoline-8-sulfonamide
2,4MP-TQS: *cis-cis*-4-(2,4-dimethylphenyl)-3a,4,5,9b-tetrahydro-3*H*-cyclopenta[c]quinoline-8-sulfonamide
2,4,5MP-TQS: *cis-cis*-4-(2,4,5-trimethylphenyl)-3a,4,5,9b-tetrahydro-3*H*-cyclopenta[c]quinoline-8-sulfonamide
2,4,6MP-TQS: *cis-trans*-4-mesityl-3a,4,5,9b-tetrahydro-3H-cyclopenta[c]quinoline-8-sulfonamide
2,5MP-TQS: *cis-cis*-4-(2,5-dimethylphenyl)-3a,4,5,9b-tetrahydro-3*H*-cyclopenta[c]quinoline-8-sulfonamide
2,6MP-TQS: *cis-trans*-4-(2,6-dimethylphenyl)-3a,4,5,9b-tetrahydro-3*H*-cyclopenta[c]quinoline-8-sulfonamide
3MP-TQS: *cis-cis*-4-*m*-tolyl-3a,4,5,9b-tetrahydro-3*H*-cyclopenta[c]quinoline-8-sulfonamide
3,4MP-TQS: *cis-cis*-4-(3,4-dimethylphenyl)-3a,4,5,9b-tetrahydro-3*H*-cyclopenta[c]quinoline-8-sulfonamide
3,4,5MP-TQS: *cis-cis*-4-(3,4,5-trimethylphenyl)-3a,4,5,9b-tetrahydro-3H-cyclopenta[c]quinoline-8-sulfonamide
3,5MP-TQS: *cis-cis*-4-(3,5-dimethylphenyl)-3a,4,5,9b-tetrahydro-3*H*-cyclopenta[c]quinoline-8-sulfonamide
4BP-TQS: *cis-cis*-4-(4-bromophenyl)-3*a*,4,5,9*b*-tetrahydro-3*H*-cyclopenta[*c*]quinoline-8-sulfonamide
4MP-TQS: *cis-cis*-4-*p*-tolyl-3a,4,5,9b-tetrahydro-3*H*-cyclopenta[c]quinoline-8-sulfonamide
ANOVA: analysis of variance
CPBG: 1-(3-chlorophenyl)biguanide hydrochloride
MLA: methyllycaconitine
NAM: negative allosteric modulator
PAM: positive allosteric modulator
PentaMP-TQS: *cis-trans*-4-(2,3,4,5,6-pentamethylphenyl)-3a,4,5,9b-tetrahydro-3*H*-cyclopenta[c]quinoline-8-sulfonamide
SAM: silent allosteric modulator
TQS: *cis-cis*-4-(napthalen-1-yl)-3*a*,4,5,9*b*-tetrahydro-3*H*-cyclopenta[*c*]quinoline-8-sulfonamide.
